# Cannabinoids in Audiogenic Seizures: From Neuronal Networks to Future Perspectives for Epilepsy Treatment

**DOI:** 10.3389/fnbeh.2021.611902

**Published:** 2021-02-11

**Authors:** Willian Lazarini-Lopes, Raquel A. Do Val-da Silva, Rui M. P. da Silva-Júnior, Alexandra O. S. Cunha, Norberto Garcia-Cairasco

**Affiliations:** ^1^Neuroscience and Behavioral Sciences Department, Ribeirão Preto School of Medicine, University of São Paulo, São Paulo, Brazil; ^2^Neurophysiology and Experimental Neuroethology Laboratory (LNNE), Physiology Department, Ribeirão Preto School of Medicine, University of São Paulo, São Paulo, Brazil; ^3^Physiology Department, Ribeirão Preto School of Medicine, University of São Paulo, São Paulo, Brazil

**Keywords:** epilepsy, audiogenic seizures, neuronal networks, cannabidiol, endocannabinoid system, CB1 receptors, *Cannabis*-derived compounds, genetically-developed strains

## Abstract

Cannabinoids and *Cannabis*-derived compounds have been receiving especial attention in the epilepsy research scenario. Pharmacological modulation of endocannabinoid system's components, like cannabinoid type 1 receptors (CB1R) and their bindings, are associated with seizures in preclinical models. CB1R expression and functionality were altered in humans and preclinical models of seizures. Additionally, *Cannabis*-derived compounds, like cannabidiol (CBD), present anticonvulsant activity in humans and in a great variety of animal models. Audiogenic seizures (AS) are induced in genetically susceptible animals by high-intensity sound stimulation. Audiogenic strains, like the Genetically Epilepsy Prone Rats, Wistar Audiogenic Rats, and Krushinsky-Molodkina, are useful tools to study epilepsy. In audiogenic susceptible animals, acute acoustic stimulation induces brainstem-dependent wild running and tonic-clonic seizures. However, during the chronic protocol of AS, the audiogenic kindling (AuK), limbic and cortical structures are recruited, and the initially brainstem-dependent seizures give rise to limbic seizures. The present study reviewed the effects of pharmacological modulation of the endocannabinoid system in audiogenic seizure susceptibility and expression. The effects of *Cannabis*-derived compounds in audiogenic seizures were also reviewed, with especial attention to CBD. CB1R activation, as well *Cannabis*-derived compounds, induced anticonvulsant effects against audiogenic seizures, but the effects of cannabinoids modulation and *Cannabis*-derived compounds still need to be verified in chronic audiogenic seizures. The effects of cannabinoids and *Cannabis*-derived compounds should be further investigated not only in audiogenic seizures, but also in epilepsy related comorbidities present in audiogenic strains, like anxiety, and depression.

## Introduction

Epilepsy is a neurological disorder characterized by the presence of epileptic seizures and their behavioral, physiological, and social consequences (Fisher et al., 2014; Kanner, 2017). Despite the great variety of antiepileptic drugs (Löscher, 2017) one third of patients remain pharmacoresistant and cannot have their seizures under control with the available pharmacological treatment (Kwan and Brodie, 2010), indicating that new therapeutic and pharmacological targets are needed. In that context, the endocannabinoid system (ES) has been receiving especial attention in the epilepsy research scenario. The ES comprises the cannabinoids receptors type 1 (CB1R) and type 2 (CB2R) and their endogenous bindings, the endocannabinoids. CB1R has been receiving especial attention in epilepsy due to seizure control in several preclinical models and can also be modulated by phytocannabinoids (Wallace et al., 2003; Lutz, 2004; Blair, 2006; Huizenga et al., 2017; Britch et al., 2020). Moreover, anticonvulsant effects were associated with *Cannabis*-derived compounds, especially the cannabidiol (CBD), reinforcing the role of cannabinoids in epileptic seizures (Friedman and Devinsky, 2015; Rosenberg et al., 2017; Lazarini-Lopes et al., 2020b). CBD, a phytocannabinoid present in *Cannabis* sp. (Jacob and Todd, 1940; Mechoulam and Shvo, 1963), has receiving especial attention due to its anticonvulsant properties in animal models of the epilepsies (Jones et al., 2012; Do Val-da Silva et al., 2017; Lazarini-Lopes et al., 2020b) and also in humans with pharmacoresistant epilepsy (Press et al., 2015; Devinsky et al., 2016, 2017). Although CBD anticonvulsant mechanisms of action seem to be related with a great diversity of cellular and molecular targets, which include components of the ES, the possible existence of synergistic effects between CBD and conventional anticonvulsant drugs may not be ignored (Mencher and Wang, 2005; Devinsky et al., 2014; Gaston et al., 2017). Additionally, although CBD has limited effects at cannabinoids receptors, CBD can modulate CB1R activity by indirect mechanisms of action (Britch et al., 2020). Therefore, the ES arise as important endogenous mechanism for seizure control (Alger, 2004; Hofmann and Frazier, 2013).

Animal models are essential for the development and screening of new anticonvulsant drugs and to evaluate their effects on the brain and on behavior (Löscher, 2011, 2017). Since epilepsies are greatly diverse in etiology, the differences between seizure induction protocols are extremely important to help understanding neuronal alterations associated with each type of seizure induction and, consequently, their clinical applications (Löscher, 2017). Audiogenic seizures (AS) are induced by intense sound stimulation (~100–120 dB) in susceptible animals and are used to study epilepsies-related mechanisms such as neuronal pathways and endogenous alterations associated with seizure susceptibility (Garcia-Cairasco et al., 2017). Audiogenic susceptible rodent strains are widely used around the world, beginning with the oldest colony, the Krushinsky-Molodkina (KM) rats in Russia (Poletaeva et al., 2017), followed by the Genetically Epilepsy-Prone Rats (GEPR) in the United States (Reigel et al., 1986; Dailey et al., 1989), the DBA/1 and DBA/2 mice (Jensen et al., 1983; Faingold et al., 2010), the Wistar Audiogenic Rat (WAR) in Brazil (Doretto et al., 2003; Garcia-Cairasco et al., 2017), among others (Ross and Coleman, 2000).

Acute AS are considered a model of generalized tonic-clonic seizures, with seizures characterized by an initial wild running phase with jumping and atonic falls followed by tonic or tonic-clonic seizures (Faingold, 1988; Terra and Garcia-Cairasco, 1992; Garcia-Cairasco et al., 1996, 2017; Ross and Coleman, 2000). However, when animals are chronically exposed to the AS protocol, called Audiogenic Kindling (AuK) (Marescaux et al., 1987), some audiogenic susceptible animals develop limbic seizures, characterized by the appearance of new behaviors such as facial and forelimb clonus, usually followed by elevation and falling (Naritoku et al., 1992; Garcia-Cairasco et al., 1996), similar to those described by Racine's scale (Racine, 1972). While brainstem sensory motor structures are primarily involved in the acute AS expression (Faingold, 1988; Terra and Garcia-Cairasco, 1992), cortical and limbic structures are associated with behavioral, EEG, and histological alterations during the AuK, indicating an expansion of the initially brainstem-dependent seizure networks to limbic regions and networks (Marescaux et al., 1987; Naritoku et al., 1992; Garcia-Cairasco et al., 1996; Moraes et al., 2000; Galvis-Alonso et al., 2004). Therefore, the AuK is as a model of temporal lobe recruitment and consequently of temporal lobe epilepsy (Moraes et al., 2000; Romcy-Pereira and Garcia-Cairasco, 2003). Other quite important characteristic is that genetic and chronic models, like susceptible strains and the AuK, can be used also to study the comorbidities, usually from neuropsychiatric origin, associated with epilepsies (Garcia-Cairasco et al., 2017).

Therefore, the purpose of the present study was to review the neuronal networks associated with AS expression. Additionally, we reviewed the effects of ES modulation and *Cannabis*-derived compounds in AS. We discussed cannabinoids modulation in AS neuronal pathways and the future perspectives of cannabinoids in AS and comorbidities.

## Neuronal Networks Involved in Audiogenic Seizures

Since AS are evoked by a high-intensity acoustic stimulus, the primary auditory pathway has been the first cluster of structures to be evaluated in audiogenic susceptible rodent strains. In this context, several research groups have detected peripheral alterations associated with AS susceptibility, such as hearing loss (Saunders et al., 1972; Glenn et al., 1980; Faingold et al., 1990), unbalance between GABAergic and Glutamatergic neurotransmissions between the inner hair cells and the cochlear nerve (Altschuler et al., 1989; Bobbin et al., 1990; Lefebvre et al., 1991), and tinnitus followed by intensity sound exposure (Heffner and Harrington, 2002; Chen et al., 2013). Similarly, anatomical and morphological alterations in the organ of Corti and in the inner and outer hair cells of the GEPRs have already been observed (Penny et al., 1983, 1986). However, despite the importance of peripheral alterations in the onset of AS, the present review will focus on brain sites involved on the onset, maintenance, and expression of AS, specifically in the brainstem (acute AS) and limbic areas (AuK).

### Brainstem Structures Critical for Acute Audiogenic Seizure Expression

#### Inferior Colliculus

It is widely accepted that inferior colliculus (IC) circuits play a pivotal role in the genesis and maintenance of sound-induced seizures (Garcia-Cairasco, 2002; Coleman et al., 2017; Ribak, 2017). The IC anatomy in the rat presents a structure similar to the human IC (Faye-Lund and Osen, 1985) and it is usually divided into the central nucleus, dorsal cortex, and external cortex (Faye-Lund and Osen, 1985; Coleman and Clerici, 1987). The central nucleus of the IC is the largest division of the IC, sends glutamatergic projections to both external cortex and dorsal cortex of the IC, and receives projections from the dorsal cortex (Coleman and Clerici, 1987; Saint Marie, 1996).

Glutamate is the main excitatory neurotransmitter into the IC and it is also implicated in the expression of AS (Faingold, 2002). Using WARs, Terra and Garcia-Cairasco (1994) showed that AP-7 administration into de central nucleus of the IC or intra-dorsal cortex of the IC, blocked or attenuated (wild runnings were still present) AS, respectively. Therefore, these intracollicular pathways may contribute to seizure propagation through its known glutamatergic connections between the external cortex of the IC and motor areas (Caicedo and Herbert, 1993; Saint Marie, 1996).

By contrast, intracollicular and extracollicular pathways are mostly modulated by GABAergic signaling (Faingold, 2002; Ribak, 2017). Therefore, deficits in GABA-mediated inhibition may be a critical mechanism associated with AS susceptibility, since a reduction in GABAergic neurotransmission in the IC was shown to facilitate neuronal firing in response to high acoustic stimuli and trigger AS (Faingold et al., 1986; Faingold, 2002).

Administration of GABA agonists into the central nucleus of the IC blocked AS expression in GEPRs and similar results were observed after pharmacological manipulations capable of increasing endogenous GABA signaling (Faingold, 2002). Administration of GABA agonists into the central nucleus of the IC blocked AS expression and the same was observed when endogenous GABA was increased in GEPRs (Faingold, 2002). Curiously, the number of GABAergic cells and the labeling of GABA synthetic enzymes are higher in GEPRs than in their Sprague-Dawley controls (Roberts et al., 1985; Ribak, 2017). However, in spite of the increased expression of all of these GABAergic biomarkers, there is a paradoxical decreased effectiveness of GABA-mediated inhibition in the IC of GEPRs (Faingold et al., 1986). Furthermore, inhibition of GABAergic neurotransmission into the IC observed in tissue slices of GEPRs (Evans et al., 2006) is thought to be the clue alteration in the triggering of AS in these animals (Faingold, 2012). Interestingly, GABA synthesis was increased in IC of KM rats, whereas GABA levels were not different from non-susceptible rats (Solius et al., 2016). It is worth to note that pharmacological activation of CB1R increased IC neuronal output, probably by activation of CB1R in GABAergic pre-synaptic terminals (Valdés-Baizabal et al., 2017). These results suggest that CB1R in the IC could play an important role on AS susceptibility.

#### Superior Colliculus

The superior colliculus (SC) is the most important non-auditory IC target (output) (Faingold, 2012). Interconnections between the external cortex of the IC and the deep layers of the superior colliculus (DLSC) seem to play an important role on AS generation and propagation (Coleman and Clerici, 1987; García del Caño et al., 2006). Since DLSC projects directly and indirectly to the spinal cord and to brainstem motor areas, such as the reticular formation (Masino and Knudsen, 1992; King et al., 1996; May, 2005), excessive activity in this network may lead to AS propagation.

Electrophysiological recordings in freely moving GEPR-9s showed increased tonic firing of DLSC neurons just prior and during the wild running, but not during the tonic behavior (Faingold and Randall, 1999). The role of DLSC in AS manifestations has already been demonstrated in WARs through their mesencephalic pathways. Midcollicular transections (knife cuts between IC and SC) blocked tonic-clonic seizures (Tsutsui et al., 1992) in WARs. Similar effects were also confirmed by Ribak et al. (1994) in GEPR-9s. Likewise, bilateral transections separating DLSC and *substantia nigra pars reticulata* (SNr) abolished tonic-clonic seizures and also attenuated wild running behaviors (Doretto et al., 2009). Browning et al. (1999) confirmed similar effects after pre-collicular transections in both GEPR substrains, GEPR-3s and GEPR-9s. Similarly, electrolytic lesions of the DLSC (but not dorsal SC) decreased AS severity in GEPRs (Merrill et al., 2003) and abolished all seizure behaviors in DBA/2 audiogenic mice (Willott and Lu, 1980).

Additionally, optogenetic activation of DLSC neurons attenuated seizures in several animal models, including AS in GEPR−3s (Soper et al., 2016). The activation of neurons in the DLSC is considered to be part of an endogenous seizure control system with origin in neurons from SNr (Gale et al., 1993; Soper et al., 2016). According to this idea, the activation of neurons from DLSC will lead to the desynchronization of epileptic brain networks (Dean et al., 1991; Soper et al., 2016). For this reasons, optogenetic stimulation of specific neurons or projections into the SC are considered an important approach to better understanding the role of the SC in AS, although the role of specific neuronal projections from SC still needs to be assessed.

#### Periaqueductal Gray Matter

Although the periaqueductal gray matter (PAG) is classically involved in emotional-related behaviors, such as fear, anxiety and panic-like behaviors (Bueno et al., 2005; Brandão et al., 2008; Deng et al., 2016), the involvement of PAG with the motor AS expression comes from findings that PAG blockade inhibits AS, more specifically the tonic and clonic behaviors, in GEPR-9s (N'Gouemo and Faingold, 1998).

Differences in the PAG neuronal firing pattern were observed in GEPRs. PAG neuronal activity increased just before the onset of the wild running, but the most remarkable neuronal tonic firing pattern was observed just prior and during the tonic behavior, but this neuronal pattern disappeared when the post-ictal depression began (N'Gouemo and Faingold, 1998). Also, the blockade of NMDA receptors or GABA_A_ activation into the PAG were both capable of suppressing AS in GEPRs, with a most potent effect associated with NMDA blockade (N'Gouemo and Faingold, 1999). In the same line, Yang et al. (2003) showed that intra-PAG AP-7 administration attenuated AS induced by ethanol withdrawal in Sprague-Dawley rats.

Classically, it has been accepted that PAG receives projections from neurons of the DLSC (King et al., 1996; Faingold et al., 2014) and projects to the sites of initiation of the motor responses associated with fight or flight reactions (Brandão et al., 1999). Additionally, there are direct and indirect (through BRF) connections between PAG and spinal cord motor neurons (Mouton and Holstege, 1994; Bajic and Proudfit, 1999) that may also contribute to AS expression. Additionally, a recent study showed that IC neurons project directly to PAG and, when optogenetically activated, triggered a sound-mediated escape response (Xiong et al., 2015). Similarly, PAG might be important not only in the acute AS, but in the kindled AS (AuK protocol). In this respect, Tupal and Faingold (2012) showed that the electrical stimulation of central nucleus of the amygdala induces intensity-dependent firing in the PAG of GEPR-9s. Additionally, GEPR-9s submitted to AuK present increased responsiveness of PAG neurons to electrical stimulation of the amygdala when compared to control GEPR-9s (Tupal and Faingold, 2012).

#### Brainstem Reticular Formation

Wada's group demonstrated for the first time the role of BRF in seizures using amygdala kindled cats, where electrolytic lesions into the BRF attenuated amygdaloid seizures and these effects were not dependent on forebrain sites (Wada and Sato, 1975).

Sprague-Dawley rats submitted to ethanol withdrawal presented increased AS susceptibility and increased spontaneous neuronal firing in the BRF, as well as increased sound-evoked activity in neurons from the same structure (Faingold and Riaz, 1994). Moreover, there is an increase in neuronal firing into the pontine nucleus of the BRF, once the AS begins and an additional increase simultaneously with the onset of the tonic seizure, that remained until the end of the tonic hind limb extension (Faingold and Randall, 1995). These data suggested that pathological conditions, like ethanol withdrawal, may induce physiological changes in the BRF, which in turn, facilitate AS expression.

Both systemic and intra-BRF NMDA antagonist administration blocked AS during ethanol withdrawal. Moreover, the increase in BRF excitatory activity was capable of inducing AS-like behaviors in previously non-susceptible rats treated with NMDA. These effects were dose-dependent, with lower dose inducing wild running behaviors and higher dose inducing wild running and generalized tonic-clonic seizures, in both cases sound-independent set of behaviors. Moreover, sound stimulation was also capable of inducing AS with wild running and generalized tonic-clonic behaviors in these animals (Ishimoto et al., 2000). In susceptible GEPR-3s, NMDA infusion into the BRF was capable of inducing seizures without the presence of acoustic stimulation (Faingold et al., 1989). On the other hand, blockade of NMDA receptors into the BRF induced a decrease in AS severity in GEPRs (Millan et al., 1988). Additionally, increased glutamate levels into the BRF had been previously observed during the tonic phase of AS in GEPRs (Chapman et al., 1986).

These data, therefore, suggest that excitatory connections between IC and BRF (Browning, 1986; Caicedo and Herbert, 1993; Riaz and Faingold, 1994) and between BRF and spinal cord (Jones and Yang, 1985) should be important efferent neuronal pathways for motor manifestation of AS (Garcia-Cairasco, 2002; Faingold et al., 2014).

#### Substantia Nigra

During the 80's, Karen Gale's group proposed that GABAergic neurotransmission into SNr should be part of what they called as an endogenous anticonvulsant system (Iadarola and Gale, 1982; Maggio and Gale, 1989). These authors proposed that a decrease in the inhibitory tonus from the SNr to the midbrain tectum might enhance seizure susceptibility.

Following up on those proposals, a series of experiments in control Wistar rats gave support to that hypothesis. Electrolytic lesions in the SNr increased AS susceptibility in Wistar rats, without any modification on locomotion, exploratory activity or grooming behaviors (Garcia Cairasco and Sabbatini, 1983; Garcia-Cairasco and Sabbatini, 1991; Garcia-Cairasco and Triviño-Santos, 1989). Nonetheless, the same SNr electrolytic lesion did not induce any alterations in AS displayed by WARs (Doretto and Garcia-Cairasco, 1995). However, neuroethological analysis based upon detailed behavioral descriptions, demonstrated changes in the behavioral structural sequence of tonic-clonic seizures in SNr-lesioned WARs. Behavioral components were present no more in a defined pattern, but randomly and fragmented, indicating that GABAergic signaling from SNr should play an important role in temporal and spatial motor integration during AS (Garcia Cairasco and Sabbatini, 1983; Doretto and Garcia-Cairasco, 1995). Curiously, it was observed that GEPRs present a disruption in the nigral GABAergic signaling, detected as a failure to release GABA from SNr, when animals were stimulated with KCl in a depolarizing protocol with microdialysis membranes into SNr (Doretto et al., 1994). These GABAergic deficits could be an explanation for the lack of seizure behavioral alteration in SNr-lesioned WARs, but, at the same time, it explains and strengthens the view that lesioned normal Wistar rats may become susceptible to AS (Garcia Cairasco and Sabbatini, 1983; Doretto and Garcia-Cairasco, 1995).

In ethanol withdrawal-induced AS, muscimol, a selective GABA_A_ agonist, applied intra-SNr reduced seizure severity during the most critical period of hyperexcitability (Gonzalez and Hettinger, 1984). Also, pharmacological activation of GABA_A_ receptors into SNr was capable of blocking AS induced by IC bicuculline injections (Terra and Garcia-Cairasco, 1992) and decreased spontaneous spike-wave discharges duration in a model of absence seizure (Depaulis et al., 1988). Conversely, specific lesions in dopaminergic neurons of the substantia nigra compacta (SNc) with the 6-OHDA toxin, an experimental model of Parkinson (Schober, 2004), did not produce AS sensitivity in resistant animals, suggesting that changes in AS susceptibility are associated with GABAergic neurons, mostly present into the SNr.

SNr projects to IC (Olazábal and Moore, 1989) and SC (Appell and Behan, 1990), regulating efferent seizure pathways (Gale, 1992). Additionally, modulation of SC by SNr may involve the neostriatum activity, which sends the main GABAergic input to SNr (Nisenbaum et al., 1992). Therefore, increasing GABA activity in the SNr is believed to be pro-convulsant because the resulting reduction of GABAergic neurotransmission into SNr-SC pathway facilitates output from SC to motor structures, such as the BRF, which may lead to seizure expression.

The findings on the so-called endogenous anticonvulsant system were supported by optogenetic inhibition of the nigrotectal terminals into the DLSC, attenuating AS in GEPR-3s. Light delivery increased the latency to the onset of AS and decreased their duration and severity in GEPR-3s (Wicker et al., 2019). These results can be explained by a decrease of GABAergic neurotransmission from SNr to the SC and are in agreement with Soper et al. (2016), who showed attenuation of AS associated with optogenetic activation of the DLSC and it is in line with the activation of SC as capable of desynchronizing cortical activity (Dean et al., 1991). In spite of this highly coherent group of studies and results, specific neurons and projections associated with the mentioned anticonvulsant effects still need to be verified *in vitro* and *in vivo*.

### Chronic Audiogenic Seizures and Neuroplastic Effects in Neuronal Networks

The repetitive audiogenic stimulus, or AuK, results in behavioral, EEG, and histological alterations in forebrain structures, such as amygdala, hippocampus, and cortex, indicating limbic recruitment (Marescaux et al., 1987; Naritoku et al., 1992; Moraes et al., 2000; Vinogradova, 2017). Marescaux et al. (1987) did behavioral observations and cortical (surface) EEG recordings in Wistar rats and proposed the term “kindling,” analogous with the limbic seizures protocols published by Goddard (1967) and Goddard et al. (1969). Naritoku et al. (1992) confirmed similar results in both GEPRs substrains: GEPR-3s and GEPR-9s, moderate and severe AS, respectively. Similar protocols and studies with quantitative behavioral methods of the evolution of AuK were made in WARs (Garcia-Cairasco et al., 1996; Galvis-Alonso et al., 2004).

The neuroanatomical and functional interaction between midbrain auditory and forebrain limbic systems can be particularly well-observed during the AuK. Local changes in the IC circuits can lead to increased collicular outputs to the limbic system, causing the seizure spread. Coupled video-EEG allowed a detailed characterization of the progression of synchronized behavior and electrophysiology with EEG recording from IC (brainstem) to hippocampus, amygdala and cortex (Moraes et al., 2000; Romcy-Pereira and Garcia-Cairasco, 2003). Furthermore, it was reported an increase in the firing rate of neurons from the central nucleus of the IC of GEPR-9s before the appearance of generalized post-tonic clonus during the AuK (N'Gouemo and Faingold, 1996). Neurons from the central nucleus of the IC project to the medial geniculate nucleus of the thalamus (MGN), as part of the primary auditory system, where they make synapsis with neurons projecting to the auditory cortex, amygdala and the hippocampus (Ledoux et al., 1985; Clugnet and LeDoux, 1990). Indeed, the amygdala and the hippocampus are the major limbic structures that receive the output from the brainstem central auditory system (Kraus and Canlon, 2012). These structures, more remarkably the amygdala, are associated with emotional context and sensorial perception, including sound stimuli (LeDoux, 2007; Kraus and Canlon, 2012).

As a clear evidence of the activation of prosencephalic structures, Simler et al. (1999) demonstrated increase c-Fos expression directly related with AuK progression: from the auditory brainstem to amygdala and perirhinal cortex, then to the frontoparietal cortex, and finally to the hippocampus and the entorhinal cortex. Simultaneous EEG recordings of IC, amygdala and auditory cortex were analyzed in WARs during AuK and it was observed that the epileptiform activity in the IC increases as AuK progresses and limbic seizures start to co-exist with brainstem seizures (Garcia-Cairasco et al., 1996; Moraes et al., 2000). Altogether, these data indicate that the progression of seizures during AuK may not be the linear expression of a simple system, but rather a complex expression of a bi-directional interaction between limbic and brainstem circuits. This is absolutely clear from the observation of a mirror (opposite) image of the decrease of brainstem-dependent seizure severity index, as soon as the AuK progress, and the increase (from zero) of the limbic-dependent seizure severity index (Garcia-Cairasco et al., 1996; Moraes et al., 2000; Rossetti et al., 2006).

Differences in hippocampus activity have been reported in audiogenic rodent strains. GABAergic currents in pyramidal neurons from CA1 of WARs are less frequent and have faster kinetics, indicating that some particular populations of interneurons might be absent in WARs (Cunha et al., 2018b). Moreover, during chronic high-intensity sound stimulation it was observed an impairment in the long-term potentiation (LTP) in non-susceptible (resistant) Wistars, but not in WARs (Cunha et al., 2015). Additionally, a decrease in the hyperpolarization activated cationic current (*Ih*) was observed in resistant animals, indicating that auditory inputs to the hippocampus might lead to compensatory homeostatic and long-term synaptic plasticity, which could be blocking the hyperexcitability of auditory pathways to the hippocampus of seizure resistant animals (Cunha et al., 2018a). In contrast, Evans et al. (1994) found different results using hippocampal slices of GEPR-9s. According to these authors, animals showed single excitatory post-synaptic potentials similarly to their control strain. However, when submitted to AuK GEPRs exhibited a more pronounced synaptic facilitation indicating that short-term potentiation is enhanced in the hippocampus of these animals (Evans et al., 1994).

## Endocannabinoid System and CB1R in Audiogenic Seizures Network

The ES is classically composed by endogenous receptors, CB1R and CB2R (Matsuda et al., 1990; Munro et al., 1993), and their endogenous ligands, anandamide, and 2-arachydonil glycerol (Devane et al., 1992; Mechoulam et al., 1995; Sugiura et al., 1995). It is widely accepted that the ES modulates neuronal activity through its retrograde action based “on-demand” endocannabinoid synthesis and release (Lutz, 2004; Alger and Kim, 2011; Castillo et al., 2012; Fitzgerald et al., 2012). However, before discussing the role of the ES modulation on seizure control in audiogenic models, it is worth to note how these cannabinoids receptors are distributed on the brain, especially on structures important to AS expression.

Autoradiography assays were used to assess CB1R distribution in several brain structures (Herkenham et al., 1990, 1991). The most intense binding was observed in the cerebellum and forebrain structures, such as several cortical and hippocampal areas. The frontal cortex presented the greater density of CB1R compared to other cortical regions, while the dorsal hippocampus seems to present more CB1R than the ventral hippocampus. The amygdaloid complex presented a moderate binding, with exception of the central nucleus that showed the lowest CB1R levels. Brainstem structures, like PAG, BRF, SC, IC, and hypothalamus presented lower levels of CB1R and sparse binding when compared to the forebrain. Like in brainstem structures, the spinal cord showed sparse binding, specifically in the dorsal horn. It is worth to note that the SNr, but not the SNc, showed the highest density levels of CB1R in the entire rat brain (Herkenham et al., 1990, 1991). Similar expression patterns were observed in others species of mammals, such as dogs, Rhesus monkeys, and humans (Herkenham et al., 1990).

Tsou et al. (1998) used immunohistochemical analysis to assess CB1R distribution in the rat brain. These authors showed CB1R in axons, dendrites, and in soma of neurons in several brain structures. Intense and widely CB1R distribution were detected in forebrain structures, such as cortical areas, as well as in amygdala, and hippocampal formation, although very restricted immunostaining were present in the brainstem, in structures like the PAG and SC. Additionally, the SNr presented a very intense immunostaining (Tsou et al., 1998), confirming those previous results observed by Herkenham's research group.

**Figure 1:**
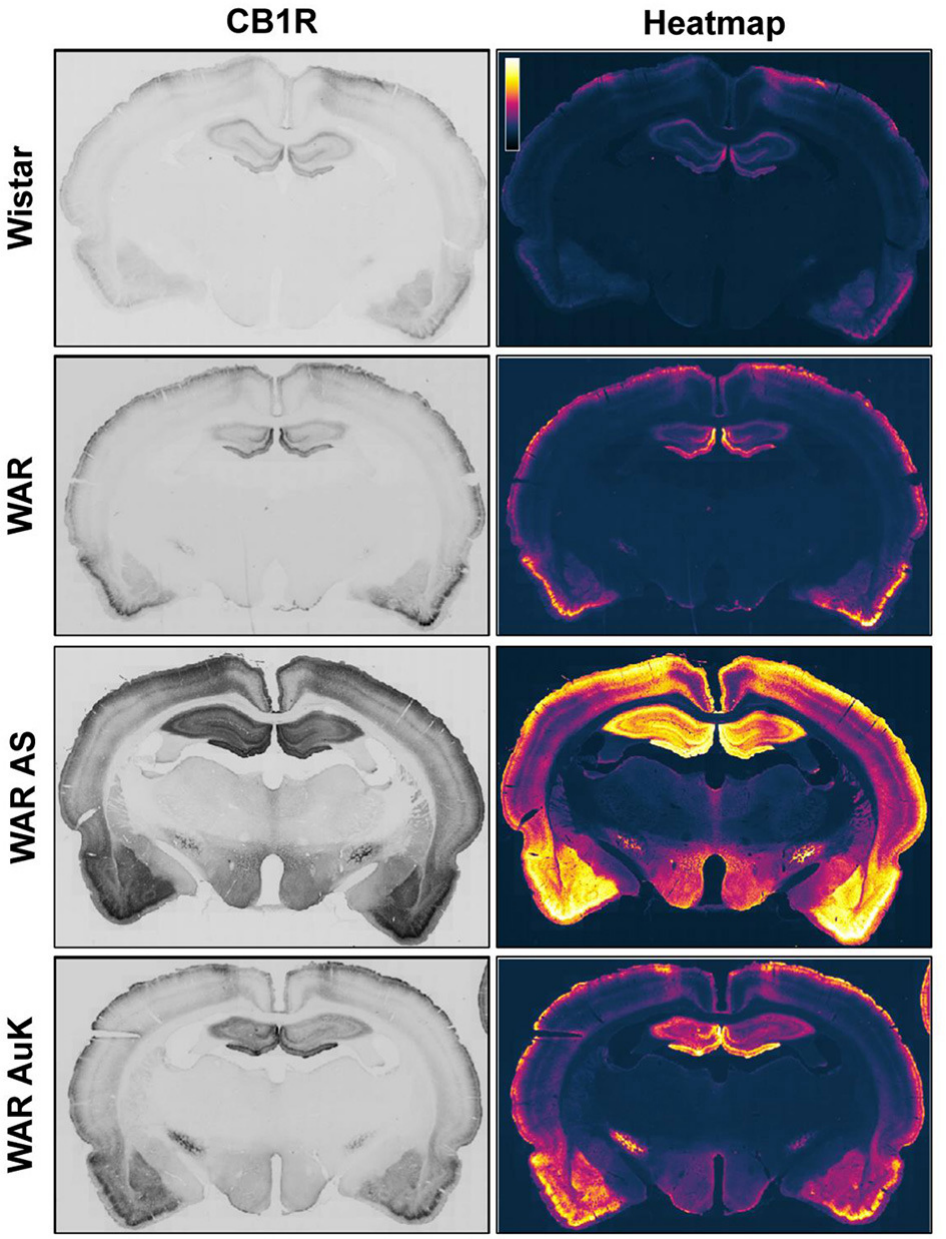
Representative CB1R immunostaining in limbic and cortical brain structures of Wistar Audiogenic Rats (WAR). It is possible to observe the increased CB1R immunostaining in limbic (hippocampus and amygdala) and cortical areas of WARs after acute audiogenic seizures (WAR AS) and chronic audiogenic seizures (WAR AuK). Wistar represents a control non-audiogenic strain. Image obtained from Lazarini-Lopes et al. (2020a).

Changes in CB1R expression and functionality have already been detected in animal models of epileptic seizures and in humans with chronic seizures (Maglóczky et al., 2010; Karlócai et al., 2011; Rocha et al., 2020). Goffin et al. (2011) assessed CB1R expression in tissue from humans with TLE and observed increased CB1R receptors expression in the seizure onset area, while CB1R expression was decreased in other areas, like the insular cortex, suggesting that different alterations in cannabinoid receptors expression could be associated with seizures expression and brain hyperexcitability (Goffin et al., 2011). However, data of CB1R expression in audiogenic strains are scarce. Increased CB1R expression was observed in the inner molecular layer of WARs, when compared to control Wistars. Additionally, in WARs, acute and chronic AS increased CB1R expression in several hippocampal layers and in specific amygdala subnuclei, the basolateral, lateral, and basomedial nuclei. Acute AS also induced changes in CB1R in the central and medial amygdala nuclei. Moreover, it is worth to note that, changes in CB1R expression in lateral, basolateral, and basomedial amygdala nuclei were correlated with limbic seizure severity during the AuK (Lazarini-Lopes et al., 2020a). See Figure 1 for a representative view of CB1R expression in limbic and cortical structures of audiogenic susceptible rats from the WAR strain.

Wistar Albino rats from Rijswijk (WAG/Rij strain) develop absence seizures along their life (van Luijtelaar and Coenen, 1986; van Luijtelaar and Sitnikova, 2006) and a subpopulation of WAG/Rij rats can also develop AS with limbic recruitment during the AuK (Vinogradova, 2008). These animals present endogenous alterations in the ES, like reduced CB1R mRNA, demonstrated by *in situ* hybridization, in the hippocampus and thalamic nuclei, brain regions associated with the genesis of absence seizures (Van Rijn et al., 2010). Therefore, further characterization of CB1R expression and functionality in brainstem and limbic sites in audiogenic WAG/Rij rats can bring important information regarding the susceptibility to AS in the WAG/Rij subpopulation.

Pharmacological CB1R activation in the intermediate layers of SC induced a robust turning behavior, these effects may be associated with modulation of GABAergic input from SNr to SC (Sañudo-Peña et al., 2000). In the SNr, CB1R are located in presynaptic terminals from the striatonigral pathways, they modulate GABA release from the nigrotectal GABAergic projections (Wallmichrath and Szabo, 2002), which may play an important role on seizure propagation and expression (Iadarola and Gale, 1982; Gale, 1986). Miller and Walker (1995) explored how the ES modulates SNr activity. WIN 55,212-2, systemically administered in normal Sprague-Dawley rats increased spontaneous firing rate in neurons from the SNr. In addition, WIN 55,212-2 also attenuated the inhibition of neuronal firing in the SNr induced by striatum electrical stimulation. In the same study, bicuculline antagonized the effects of striatum stimulation, suggesting that WIN 55,212-2 effects on SNr activity were dependent on GABAergic neurotransmission (Miller and Walker, 1995), although specific neurons and projections associated with these effects still need to be verified. Therefore, the GABAergic signaling from SNr to mesencephalic tectum may be, somehow, enhanced by cannabinoids administration, increasing the inhibitory tonus generated by this endogenous anticonvulsant system. Although, this hypothesis still needs to be further elucidated, measuring GABA release and also CB1R activity in the SNr.

**Figure 2:**
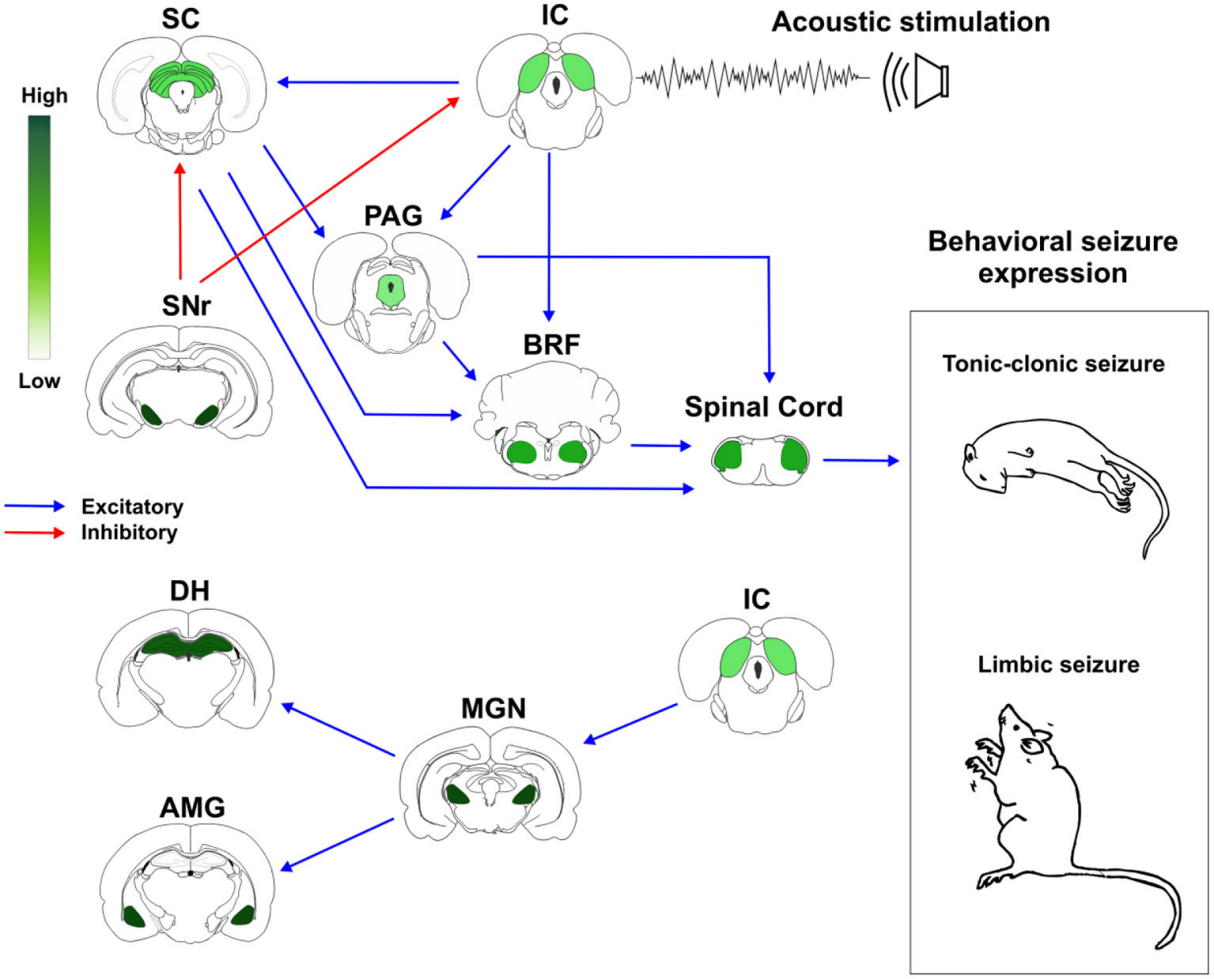
Cannabinoid receptors type 1 (CB1R) in neuronal networks associated with acute and chronic audiogenic seizures. The inferior colliculus is the main brainstem structure related to sound perception and plays a key role in the genesis of audiogenic seizures. Inferior colliculus projects to different brainstem areas, like superior colliculus, periaqueductal gray matter, and brainstem reticular formation. This brainstem neuronal network is crucial to acute audiogenic seizures manifestation, that are behaviorally characterized by wild running followed by tonic-clonic seizures. The substantia nigra pars reticulata sends GABAergic projections to the mesencephalic tectum. This inhibitory projection is involved with the so-called endogenous anticonvulsant system. During the audiogenic kindling protocol, the chronic seizures, and the epileptogenic events lead to forebrain/limbic recruitment. The limbic recruitment during the chronic seizures involves projections from inferior colliculus to medial geniculate nucleus and then to the dorsal hippocampus and basolateral amygdala nucleus. This brainstem-limbic network is crucial to limbic motor seizures expression during the audiogenic kindling. Spinal cord neurons receive inputs from central neuronal networks and lead to audiogenic seizures' motor manifestation. The intensity of green color represents the amount of CB1R in each structure. Therefore, the endocannabinoid system is directly associated with brainstem and limbic neuronal networks responsible for tonic-clonic and limbic audiogenic seizures manifestation. IC, inferior colliculus; SC, superior colliculus; PAG, periaqueductal gray matter; BRF, brainstem reticular formation; SNr, substantia nigra pars reticulata; MGN, medial geniculate body; DH, dorsal hippocampus; AMG, amygdaloid complex (basolateral amygdala nucleus, BLA). Red arrows represent inhibitory projections, blue arrows represent excitatory projections.

Based on these previous evidences, we proposed a schematic representation of how brainstem and forebrain structures with different distribution of CB1R might modulate AS susceptibility and expression (Figure 2).

## Endocannabinoid System Modulation in Audiogenic Seizures

Although cannabinoids induce modulation have already been shown as capable of epileptic seizures in several animal models (Rosenberg et al., 2017; Lazarini-Lopes et al., 2020b), studies evaluating the role of the ES on AS experimental models are still scarce. See Table 1 for main results from the literature.

**Table 1:** Effects of cannabinoids modulation and *Cannabis*-derived compounds in audiogenic seizures.

**References**	**Subjects**	**Treatment/manipulation**	**Results**
Carlini et al. (1973)	Male mice Male rats	CBD (10, 50, and 200 mg/kg; i.p.), CBD (25 mg/kg; i.p.)	Reduced wild running and tonic-clonic seizures
Boggan et al. (1973)	C57BL/6 mice submitted to AS priming	THC (2.5, 5, and 10 mg/kg; i.p.)	Reduced wild running, tonic, and clonic seizure
Consroe and Wolkin (1977)	Adult male rats	CBD (17 mg/kg; orally)	Reduced tonic-clonic seizures
Consroe et al. (1981)	Adult male rats	CBD (82.4 mg/kg; i.p.), CBD (14.9 mg/kg; i.v.)	Reduced AS frequency
Samineni et al. (2011)	Male and Female GEPR-9s	AM 251 (50, 100, and 200 pmol; intra-ventrolateral PAG; bilateral)	Decreased post-ictal analgesia (AS was not assessed)
Vinogradova et al. (2011)	Wistar rats susceptible to AS	SR 141716 (30 mg/kg; i.p.)	Facilitated AS in resistant animals; increased AS duration; facilitated limbic seizure expression
Busquets-Garcia et al. (2013)	*Fmr1–/y* mice: Fragile X Syndrome model	SR 141716 (1 mg/kg; i.p.) AM 630 (1 mg/kg; i.p.)	Attenuated AS and anxiety-like behavior
Hill et al. (2012)	DBA/2 mice	CBDV (50, 100, and 200 mg/kg; i.p.)	Reduced tonic seizures; Reduced mortality; increased the number of seizure-free animals
Hill et al. (2013)	DBA/2 mice	*Cannabis*-derived compounds rich in CBD + CBDV (Several doses)	Reduced wild running, tonic, and clonic seizures
Vinogradova and Van Rijn (2015)	Adult male KMs	WIN 55,212-2 (4 mg/kg; s.c.)	Increased the latency to post-tonic clonus behaviors
Citraro et al. (2016)	DBA/2 mice	Cannabinoid agonists: N-palmitoylethanolamine (5–40 mg/kg; i.p.), Arachidonyl-2-chloroethylamide (0.5–30 mg/kg; i.p.), WIN 55,212-2 (2.5–60 mg/kg; i.p.)	All drugs attenuated AS. Anticonvulsant effects were antagonized by NIDA-41020 (0.5–2 mg/kg; i.p.), a CB1R antagonist.
Gu et al. (2019)	Angelman Syndrome mice susceptible to AS	CBD (10, 20, 50, and 100 mg/kg; i.p.)	Attenuated wild running and tonic-clonic seizures. More than 80% of mice were seizure-free with CBD 100 mg/kg
Santos et al. (2020)	Male GEPR-3s	WIN 55,212-2 (1, 1.5, 2 mg/kg; i.p.), CP 55940 (26.5 nmol; intra-DLSC, bilateral)	Systemic and intra-DLSC CB1R activation attenuated AS severity; Systemic effect was not modified by intra-DLSC CB1R antagonist
Lazarini-Lopes et al. (2020a)	Male WARs	Characterization of CB1R expression after acute and chronic AS	Increased CB1R in the hippocampus of WAR (WAR × Wistar); Acute and chronic AS increased CB1R in amygdala and hippocampus of WARs

Vinogradova et al. (2011) showed that acute and chronic treatment with SR141716, a CB1R antagonist, presented pro-epileptic effects in audiogenic Wistar rats and facilitated the AuK progression. These authors reported that acute treatment with SR141716 in susceptible rats increased AS duration and severity and induced the appearance of limbic seizures behaviors. Interestingly, the treatment with the CB1R antagonist did not modify AS susceptibility in normal rats, but in susceptible animals that developed resistance to AS, the seizures reappeared after SR141716 administration (Vinogradova et al., 2011). It is worth to notice that similar results were observed in a patient that was free of seizures for more than 20 years, but after administration of CB1R antagonist for obesity treatment the seizures reappeared. In that case, when the CB1R antagonist treatment was interrupted, the seizures disappeared (Braakham et al., 2009).

Conversely, a single administration of WIN 55,212-2, a CB1R agonist, presented long-term, but not acute effects, against AS, increasing the latency to the onset of post-tonic clonus in KM rats (Vinogradova and Van Rijn, 2015). Moreover, pharmacological treatments with different cannabinoid receptors agonists were capable of attenuating AS in DBA/2 mice, reducing wild running, clonus, and tonus behaviors. These anticonvulsant effects were blocked by previous administration of NIDA-41020, a selective CB1R antagonist (Citraro et al., 2016). Additionally, these authors also demonstrated that when ineffective doses of cannabinoid receptors agonists were co-administered with classical anticonvulsant drugs, such as carbamazepine, gabapentin, phenobarbital, and valproate, the anticonvulsant effects of all these drugs were potentiated (Citraro et al., 2016). In GEPR-3s, systemic administration of WIN 55,212-2, was effective against AS, suppressing seizures in 9/10 animals and attenuating seizure severity in 1/10 rats. Similarly, central administration of CP 55940, a CB1/2 agonist, directly into the DLSC of GEPR-3s, suppressed seizures in 6/9 rats, reduced seizure severity in 2/9, and had no effect in 1/9. Additionally, intra-DLSC administration of SR141716 did not modify AS in control GEPRs and did not antagonize the anticonvulsant effects induced by systemic WIN 55,212-2 (Santos et al., 2020). Also using GEPRs, Samineni et al. (2011) showed that central injection of AM251, a CB1R antagonist, directly into the ventrolateral PAG, attenuated post-ictal analgesia in GEPR-9s. These data suggest that AS results in increased endocannabinoid levels in the PAG, which may mediate post-ictal analgesia (Samineni et al., 2011). Since the last study did not look at seizure expression after CB1R modulation, it should be interesting to assess the role of CB1R from PAG in AS expression in GEPRs and other audiogenic strains.

Using the *Fmr1* knockout mice to mimic the fragile X syndrome, authors observed that pharmacological blockade of CB1R rescued several pathological alterations, including the increased susceptibility to AS. Additionally, blockade of CB2R also induced anxiolytic behavior in the elevated plus maze (Busquets-Garcia et al., 2013). In the WAG/Rij strain, a model of absence seizures with a subpopulation also susceptible to AS, systemic administration of WIN 55,212-2 reduced the number of spontaneous spike-wave discharges, but increased seizure duration, in WAG/Rijs, whereas administration of AM251 attenuated the effects of CB1R activation (Van Rijn et al., 2010). Reduced number and duration of spike-wave discharges were observed after central (intra-thalamic nucleus) anandamide or WIN 55,212-2 administration (Citraro et al., 2013). However, it is unclear if the pharmacological modulation of CB1R, or endocannabinoids, can attenuate AS in the WAG/Rij strain and the exploration of this research field, assessing acute and chronic AS in WAG/Rij rats, can bring important information and insights about ES functionality in two different types of seizures (absence and audiogenic) in the same strain.

## *Cannabis*-Derived Compounds in Audiogenic Seizures

After 40 years of the demonstration of CBD as anticonvulsant in humans (Cunha et al., 1980), the interest in medical *Cannabis*-derived compounds, especially CBD, has substantially increased, as an alternative treatment for pharmacoresistant epilepsy (Porter and Jacobson, 2013; Press et al., 2015; Devinsky et al., 2016, 2017). In 2018, the United States Food and Drug Administration (FDA) approved its first *Cannabis*-derived drug, the Epidiolex, a highly purified CBD oil solution (Corroon and Kight, 2018). This compound presents important therapeutic effect, especially in treatment-resistant epilepsy (Devinsky et al., 2018; Hausman-Kedem et al., 2018). However, although some studies have already demonstrated significant effects of Epidiolex against seizures, longitudinal studies to investigate long-term efficacy and safety are necessary, especially to assess its effects on cognitive and hormonal functions after chronic administration (Sekar and Pack, 2019).

In the basic research, CBD exerts not only anticonvulsant effects (Gobira et al., 2015; Kaplan et al., 2017; Klein et al., 2017; Lazarini-Lopes et al., 2020b), but also presents additional prominent effects important for epilepsy treatment, such as neuroprotective (Campos et al., 2016; Do Val-da Silva et al., 2017) and anti-inflammatory effects (Costa et al., 2004; Esposito et al., 2011). Because the epilepsies usually are accompanied by neuropsychiatric comorbidities, it is also important to know that CBD has antipsychotic, anxiolytic, and antidepressant effects (Zuardi et al., 1991; Crippa et al., 2011; Linge et al., 2016). As previously reported for pharmacological modulation of cannabinoid receptors, studies regarding *Cannabis*-derived compounds in AS are limited. See Table 1 for main results.

CBD anticonvulsant effects against AS were firstly demonstrated by Carlini's research group during the 70's, in Brazil, when audiogenic susceptible Wistar rats were treated with CBD and then exposed to high-intensity sound stimulation. CBD drastically reduced the incidence of AS, decreasing the expression of wild running followed by tonic-clonic seizures from 60 to 10% after an acute acoustic stimulation (Carlini et al., 1973). Using audiogenic susceptible rats, Consroe and Wolkin (1977), evaluated CBD effects in a great variety of epileptic seizure models, including AS. After three consecutive screenings for AS to confirm seizure susceptibility, CBD treatment was capable of preventing AS in a posterior stimulus. Moreover, the same research group showed that not only CBD, but also its analogs, prevented AS in 70% of animals after intravenous administration (Consroe et al., 1981). However, these studies did not discuss additional information regarding behavioral seizure profile or brain sites associated with CBD effects.

Cannabidivarin (CBDV), a CBD analog, presented dose-dependent protective effects against AS in DBA/2 mice, reducing the percentage of animals that developed tonic seizures, dropping to zero the mortality, and increasing the number of animals seizure-free (Hill et al., 2012). Similarly, *Cannabis*-derived botanical drug compounds rich in CBD were capable of reducing clonic seizures, and the co-administration of CBD and CBDV had synergic effects against generalized AS in DBA/2 mice, reducing wild running and clonic behaviors and blocking tonic seizures. This result is particularly interesting because although the authors confirmed CBD protective effects against AS, CBD anticonvulsant effects were independent of CB1R mechanisms (Hill et al., 2013). Gu et al. (2019) investigated CBD effects against AS in an animal model of Angelman Syndrome. In this model, mice are susceptible to AS, expressing wild running and tonic-clonic behaviors in response to intense sound stimulation (125 dB). CBD pretreatment presented dose-response effect, attenuating seizure expression, blocking tonic-clonic behaviors, and preventing seizure behaviors in more than 80% of Angelman Syndrome-mice tested (Gu et al., 2019). Furthermore, (–) Δ^9^-tetrahydrocannabinol (THC) protect animals against AS. Authors observed a dose-dependent effect of THC, attenuating wild running, tonic, and clonic seizures (Boggan et al., 1973).

The current data about *Cannabis*-derived compounds on AS, especially CBD, CBDV, and THC, are convergent, suggesting attenuation of wild running and tonic-clonic behaviors in acute AS. Nevertheless, cannabinoids in the context of chronic AS still need to be explored. Chronic seizure protocols, like the AuK, allow the study of drugs with anticonvulsant effects and potential antiepileptogenic effects associated with seizures progression during the chronic protocol (Simonato et al., 2014). Therefore, exploration of this research field using audiogenic strains could bring important information, especially regarding cannabinoids and the epileptogenic process.

## Conclusion and Future Perspectives

Although with some paradoxical puzzling data, AS susceptibility in several of the mentioned networks converge to a more hyperexcitable brainstem state, either by an increase in glutamatergic neurotransmission or by a decrease in GABAergic signaling. Intra-collicular circuits receive and integrate information and send projections to extra-collicular afferent and efferent pathways and excessive excitatory activity into IC, SC, PAG, and BRF seems to be related with AS expression. On the other hand, GABA signaling into SNr is believed to be part of an endogenous anticonvulsant system that seems to be modulated by the ES, especially by CB1R, which could be an important clue to explain the neuronal basis of cannabinoids effects in AS.

Genetic strains used to study AS present advantages over chemical models, such as pilocarpine- and kainic acid-induced *Status Epilepticus*, where extended lesions are displayed by the animals (Leite et al., 2002; Castro et al., 2011; Furtado et al., 2011), but the neuroplasticity in AS is not accompanied by huge structural abnormalities (Galvis-Alonso et al., 2004). Other important characteristic of genetic models of AS is the absence of possible pharmacological interaction between cannabinoids and convulsant drugs. The AS model does not require previous invasive protocols for seizure induction (i.e., stereotaxic surgery or drug administration) and the trigger is an external high intensity stimulus directly controlled by the researcher. Because of these advantages, audiogenic strains seem to be interesting and appropriate approaches for epilepsies studies (Faingold et al., 2014).

Treatment with CB1R agonists, as well as with *Cannabis*-derived compounds, like CBD and CBDV, presented anticonvulsant activity against acute AS. The most prominent effects are associated with tonic behaviors, but wild running and clonus were also attenuated by these treatments. CB1R location in brainstem and forebrain structures also supports ES modulation on AS, especially into the SNr, amygdala, hippocampus, and cortex, the brain sites with most intense CB1R expression. Although there is a lack of studies investigating CBD and ES on chronic seizures in protocols like the AuK, the current data suggest that this is a prominent research area of study for epilepsies treatment. Since chronic protocols of AS allow the study of the epileptogenic process, the AuK could be an interesting tool to assess the role of the ES and *Cannabis*-derived compounds on limbic and forebrain recruitment.

Moreover, CBD anticonvulsant effects are associated with a great variety of mechanisms of action, such as GPR55, TRPV1, 5-HT_1A_, BK channels, increased GABAergic neurotransmission, changes in calcium signaling, an indirect modulation of CB1R (Devinsky et al., 2014; Britch et al., 2020; Lazarini-Lopes et al., 2020b). In that context, antagonism of TRPV1 receptors suppressed AS in female GEPR-3s and attenuated seizures in male GEPR-3s (Cho et al., 2018). Therefore, the exploration of these mechanisms associated with cannabinoids in audiogenic strains is an interesting approach that should be further investigated. Furthermore, recent clinical data indicate that, regardless of the CBD low affinity for 5-HT1A receptors, at high concentration, CBD reduced the constitutive activity of receptors coupled to Gi/o receptors and these effects were reversed in the presence of 5-HT_1A_ antagonist, suggesting that CBD can act as a 5-HT_1A_ inverse agonist (Martínez-Aguirre et al., 2020). Therefore, the linking behind CBD anticonvulsant effects and the serotonergic system, should be further investigated in audiogenic strains.

Recent data showed that CBD attenuated seizures and restored the impaired hippocampal GABAergic neurotransmission observed in an animal model of Dravet-Syndrome (Kaplan et al., 2017). Likewise, audiogenic susceptible animals from the WAR strain present reduced GABAergic activity in the hippocampus (Cunha et al., 2018b) and the evaluation of CBD effects at GABAergic hippocampal network of WARs and other audiogenic strains could bring important information regarding CBD effects on epileptogenic process. Similarly, characterization of endocannabinoids levels and CB1R expression and functionality in brainstem and forebrain networks could help to explain the susceptibility to brainstem and limbic seizures in audiogenic strains. Data about CB1R expression in audiogenic susceptible strains are still very limited, but the current data suggest that endogenous alterations in CB1R could be related with seizure susceptibility, corroborating clinical data. Moreover, chronic alcohol exposure impairs CB1R functionality in the basolateral amygdala nucleus, which in turn, affects GABAergic signaling in this structure (Varodayan et al., 2016). Therefore, it should be an interesting approach to investigate changes in endocannabinoids levels and functionality in AS induced by ethanol withdrawal.

Finally, CBD presents different physiological and pharmacological mechanisms of action associated with its anticonvulsant effects and improvement of epilepsy-related neuropsychiatric comorbidities in basic and clinical research (Bergamaschi et al., 2011; Devinsky et al., 2014; Campos et al., 2017; Patra et al., 2020). Anxiety- and depressive-like behaviors are associated with genetic predisposition to seizure in strains like WAR, GEPR, KM, and audiogenic-susceptible WAG/Rij rats (Sarkisova and Kulikov, 2006; Castro et al., 2017; Sarkisova et al., 2017; Aguilar et al., 2018). However, the assessment of cannabinoids in epilepsy related comorbidities is an under-explored research field. Therefore, the ES in brainstem and limbic structures should be investigated not only in seizure susceptibility and expression, but also in neuropsychiatric comorbidities related to epilepsies.

## Author Contributions

WL-L, RD, and NG-C conceived the original idea. WL-L wrote the manuscript. RD, AC, RS-J, and NG-C provided critical reviews, and assisted in the writing and background research. RS-J and WL-L prepared the figures. WL-L prepared the table. NG-C reviewed the manuscript and included additional recommendations. All authors approved the final version.

## Conflict of Interest

The authors declare that the research was conducted in the absence of any commercial or financial relationships that could be construed as a potential conflict of interest.
